# Isolation of circulating tumor cells

**DOI:** 10.1016/j.isci.2022.104696

**Published:** 2022-07-01

**Authors:** Jon F. Edd, Avanish Mishra, Kyle C. Smith, Ravi Kapur, Shyamala Maheswaran, Daniel A. Haber, Mehmet Toner

**Affiliations:** 1 BioMEMS Resource Center, Center for Engineering in Medicine and Surgical Services, Massachusetts General Hospital and Harvard Medical School, Boston, MA 02114, USA; 2 Cancer Center, Massachusetts General Hospital, Boston, MA 02114, USA; 3 BendBio, Inc., Sharon, MA 02067, USA; 4 Harvard Medical School, Boston, MA 02115, USA; 5 Howard Hughes Medical Institute, Bethesda, MD 20815, USA; 6 Shriners Hospitals for Children, Boston, MA 02114, USA

**Keywords:** Biotechnology, Cell biology, Technical aspects of cell biology

## Abstract

Circulating tumor cells (CTCs) enter the vasculature from solid tumors and disseminate widely to initiate metastases. Mining the metastatic-enriched molecular signatures of CTCs before, during, and after treatment holds unique potential in personalized oncology. Their extreme rarity, however, requires isolation from large blood volumes at high yield and purity, yet they overlap leukocytes in size and other biophysical properties. Additionally, many CTCs lack EpCAM that underlies much of affinity-based capture, complicating their separation from blood. Here, we provide a comprehensive introduction of CTC isolation technology, by analyzing key separation modes and integrated isolation strategies. Attention is focused on recent progress in microfluidics, where an accelerating evolution is occurring in high-throughput sorting of cells along multiple dimensions.

## Highlights

• Circulating tumor cells (CTCs) spread cancer through the bloodstream (metastasis)
• CTC-based liquid biopsy enables minimally invasive sampling of cancer cells in blood
• Their extreme rarity requires all CTC types to be enriched from large blood volumes
• CTC isolation technology is analyzed, with a focus on high-throughput microfluidics

## Graphical abstract

**Figure:**
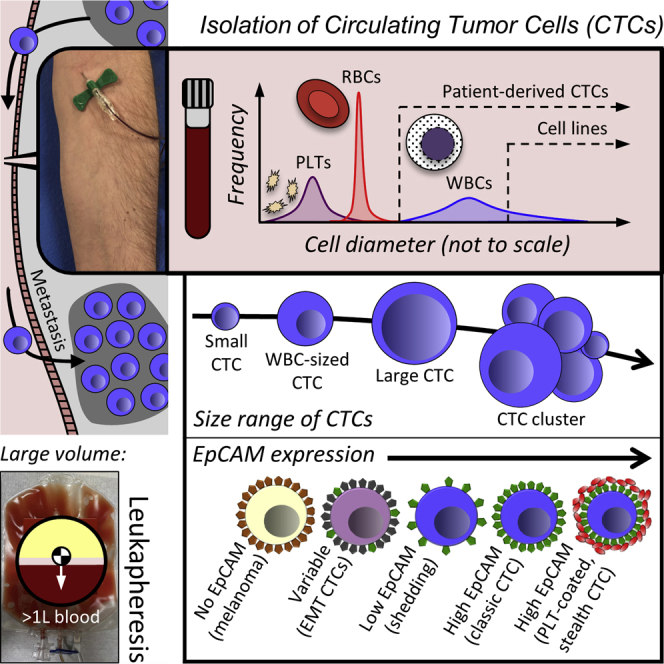

## Introduction

**Figure 1:**
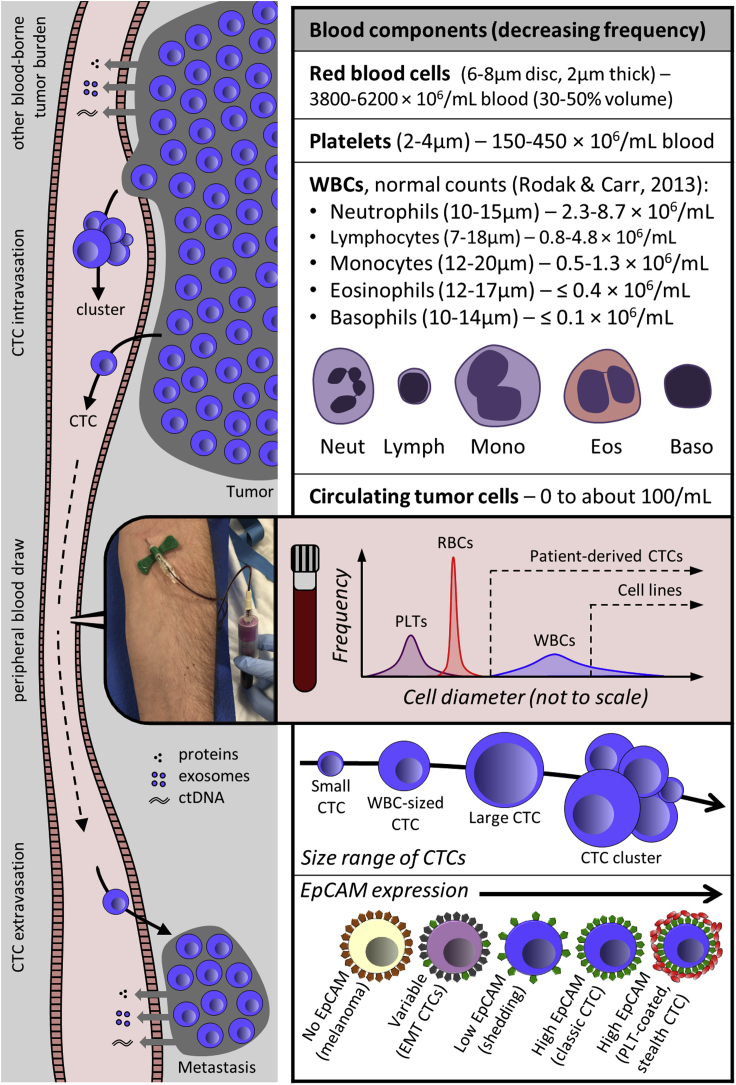
The setting and challenges of CTC isolation technology Left: alongside tumor-derived proteins, exosomes and ctDNA, cancers shed cells and clusters sporadically into the blood. Provided they are not removed from the circulation too quickly, CTCs can be isolated from peripheral circulation by venous blood draw, but their extreme rarity requires high-yield enrichment from large volumes of blood, and thus multi-log_10_ removal of normal cells (top right). Isolation is further complicated by an overlap in size of patient-derived CTCs with WBCs (sizes from blood smear) and by the variability (or lack) of available EpCAM for positive selection (lower right).

Circulating tumor cells (CTCs) originate in solid tumors and then enter the blood stream directly, by crossing the endothelium (intravasation, see Figure 1), or indirectly through the lymphatic system (Hanahan and Weinberg, 2011; Riggi et al., 2018). It has long been surmised that CTCs are the primary route by which cancer metastasizes far from its origin (Aceto et al., 2015). As such, they are a key factor in the course of disease, and their presence in the peripheral blood enables a convenient liquid biopsy of cancer.

Although circulating tumor DNA (ctDNA) (Cescon et al., 2020; Ignatiadis et al., 2021) and exosomes (Reátegui et al., 2018; Yu et al., 2021) also provide sources of tumor-specific nucleic acids in the blood that can be used for detection and genotyping, CTCs provide unique possibilities (Haber and Velculescu, 2014). Notably, personalized *in vitro* drug testing is only possible with viable intact cells, and this has been demonstrated by generating CTC-derived cell lines (Yu et al., 2014). In addition, monitoring tumor heterogeneity (Barteneva et al., 2013), as visualized by immune fluorescence (IF) or encoded in the set of mutations and gene expression profiles of multiple clonal and sub-clonal populations, requires intact cells (Hong et al., 2018; Jordan et al., 2016; Kwan et al., 2018). Therefore, considerable effort has been devoted to improving the methods for isolating CTCs from blood so that their unique and rich biological information can be put to greatest use in the multi-armed war on cancer. However, CTCs are few in number, rarely exceeding 100 cells per vacutainer, defined here as 7.5 mL blood. As summarized in Figure 1, the challenges of isolating and detecting these few CTCs with high sensitivity and specificity from among the 40 billion or more blood cells in a tube of blood can be daunting.

CTC isolation technology can trace its roots at least as far back as observations of cells in the blood of a deceased patient resembling those of the tumor (Ashworth, 1869). A logical next step was to find these cells in the blood of patients with cancer, but development of isolation technology seems to have been delayed until subsequent observations of CTCs were reported nearly 90 years later (Engell, 1955). Soon after, nascent isolation strategies arose combining centrifugation, lysis, filtration, and even nonspecific magnetic removal of phagocytes (Kuper et al., 1961). Later, positive selection became possible by targeting CTCs expressing epithelial cell adhesion molecule (EpCAM) for positive selection. The pace of technological innovation has since quickened as the unique capabilities of microfluidics have been applied to CTC isolation (Nagrath et al., 2007) using multiple separation modes and antigens, including CD45 exploited for negative selection (Ozkumur et al., 2013). Moreover, the utility of CTCs as a liquid biopsy of cancer is expanding in lockstep with the molecular biology toolkit, providing ever more information from each CTC (Micalizzi et al., 2017).

This text aims to provide a primer for those who are new to CTC isolation and desire to either develop improved isolation tools and strategies or to select an existing technology to address specific clinical applications or research questions. It is not a review in the strict sense, and many important contributions have been neglected to allow a compact discussion of the diverse array of CTC isolation strategies that have been demonstrated. The reader is referred to reviews (Cho et al., 2018; Haber and Velculescu, 2014; Ignatiadis et al., 2021; Micalizzi et al., 2017; Parkinson et al., 2012) for a more comprehensive accounting of the field.

### On the rarity of CTCs

Firstly, to understand how CTC rarity constrains isolation technology, consider a simple model for the expected CTCs in a sample volume (*v*) of total blood supply (*V*). If *β* reflects the total CTCs entering the circulation per second and *τ* is their average lifetime before removal from peripheral circulation (by destruction, entrapment in the capillaries, or extravasation), the expected number of CTCs (*λ*) is equal to *βτv/V*. Although *β* and *τ* are difficult to measure and vary widely with cancer type, stage, and in response to surgery and treatment, their product may be estimated from observed CTC numbers. In the human circulatory system, *τ* was observed in one case to be less than 24 h based on a precipitous drop in CTC counts one day after tumor resection (S.L. Stott et al., 2010a). In mice, CTC kinetics and intravasation rate were studied by exchanging blood between a tumor-bearing mouse and a healthy mouse (Hamza et al., 2021), employing two optofluidic CTC counters (Hamza et al., 2019). The authors also showed that intravasation rate varied by a few orders of magnitude (60 to 107,000 CTCs/hr) depending on cancer model and tumor stage, but variability in CTC lifetime was lower (40 to 260 s) (Hamza et al., 2021). Interestingly, Aceto et al. estimated a lifetime of 6–10 min for CTC clusters and 25–30 min for single CTCs (Aceto et al., 2014). If we assume for simplicity a fixed *τ* of 1 h, finding ten CTCs in a standard tube of blood would yield *β* = 1.9 Hz in our hypothetical case (two detectable CTCs entering the normal 5 L of peripheral blood every second).

There might of course be many CTCs that never enter an accessible vein. This possibility can be illustrated by first tracing the shortest transit of the bloodstream by a sampled CTC. Let us proceed backwards to the CTC-shedding tumor, starting from the typical phlebotomy site (a vein in the antecubital fossa), and moving upstream. This leads first to the capillary beds in the fingers and arm, where some CTCs can be removed from the blood. The path continues back through the arterial system to the aorta and left side of the heart, and then to the capillary beds in the lungs, which may also trap CTCs (or shed them from a lung tumor or metastasis). Continuing upstream, we pass through the right side of the heart and finally back to the tumor-draining vasculature, where CTC concentrations may be significantly higher. However, the fractions of CTCs which might be sequestered by each capillary bed remain largely a mystery. This is in part due to the invasive sampling necessary to observe these compartments, but also to the possibility for CTCs to make multiple transits through the circulation—even some CTC clusters are thought capable of squeezing past capillary beds with sufficient time (Au et al., 2016). Regardless, metastases are expected to originate from CTCs or clusters that become trapped and/or extravasate in any of the diverse capillary beds that feed from the arterial circulation (or in the lungs). Either compartment is upstream of blood sampling, so CTC numbers in peripheral blood might well underestimate CTC burden overall.

For metastatic patients, typical counts in the peripheral blood range from one CTC to a few hundred per vacutainer, rarely more; however, samples from one tube still sometimes result in zero CTCs being observed. The *Poisson* statistics of sampling can explain many false negatives. Specifically, the probability of finding *k* CTCs in a sample volume (*v*) may be estimated as *P*_*k*_ *= (ελ)*^*k*^ *exp(-ελ)/k!*, where ε represents CTC yield into product, and *λ* is again the expected number of CTCs in *v*. Thus, if there are on average 2 CTCs in a tube of blood, and the isolation yield is 95% (with 100% sensitivity of the downstream assay), one can expect a 15% false negative rate and just a single CTC in the tube 28.4% of the time. Taken together with the desired sensitivity (fraction of tests that provide the desired CTC-specific information above a suitably chosen specificity threshold), the scarcity of CTCs in a given cohort sets a minimum sample volume. This becomes critical for pushing detection earlier. Although CTCs were reported to exist occasionally in 10 mL blood samples from patients with COPD later diagnosed with lung cancer (Ilie et al., 2014), 100-fold fewer CTCs were surmised to be present in primary breast cancer before surgery vs. metastatic disease (Coumans et al., 2013), so hundreds of milliliters of blood, or even continuous monitoring of the circulation, may ultimately be required to get a reliable signal in cancer’s early stages. However, background interference to downstream assays also scales linearly with sample. Therefore, CTC enrichment methods must be designed to allow adequate signal (CTCs) to noise (impurities) for the chosen blood volume.

## Label-free CTC enrichment

Technologies for CTC isolation from whole blood are organized along dimensions of separability that highlight differences between some or all CTCs and the normal components of blood. To remove CTCs from the plasma, platelets, and red blood cells (RBCs), biophysical differences are sufficient for enrichment of essentially all CTCs (with the exception of any platelets that satellite onto CTCs). To separate CTCs from WBCs, biophysical properties, such as size, deformability, density, and membrane capacitance, have been used. Although technologies that leverage biomolecular differences between CTCs and WBCs have in principle a greater specificity, and thus enable higher yield and purity across the broad swath of CTCs (Figure 1), purely biophysical methods of CTC enrichment can be simpler and lower in cost. Hence, these separation modes will be discussed first.

### Bulk removal of red blood cells and platelets

**Figure 2:**
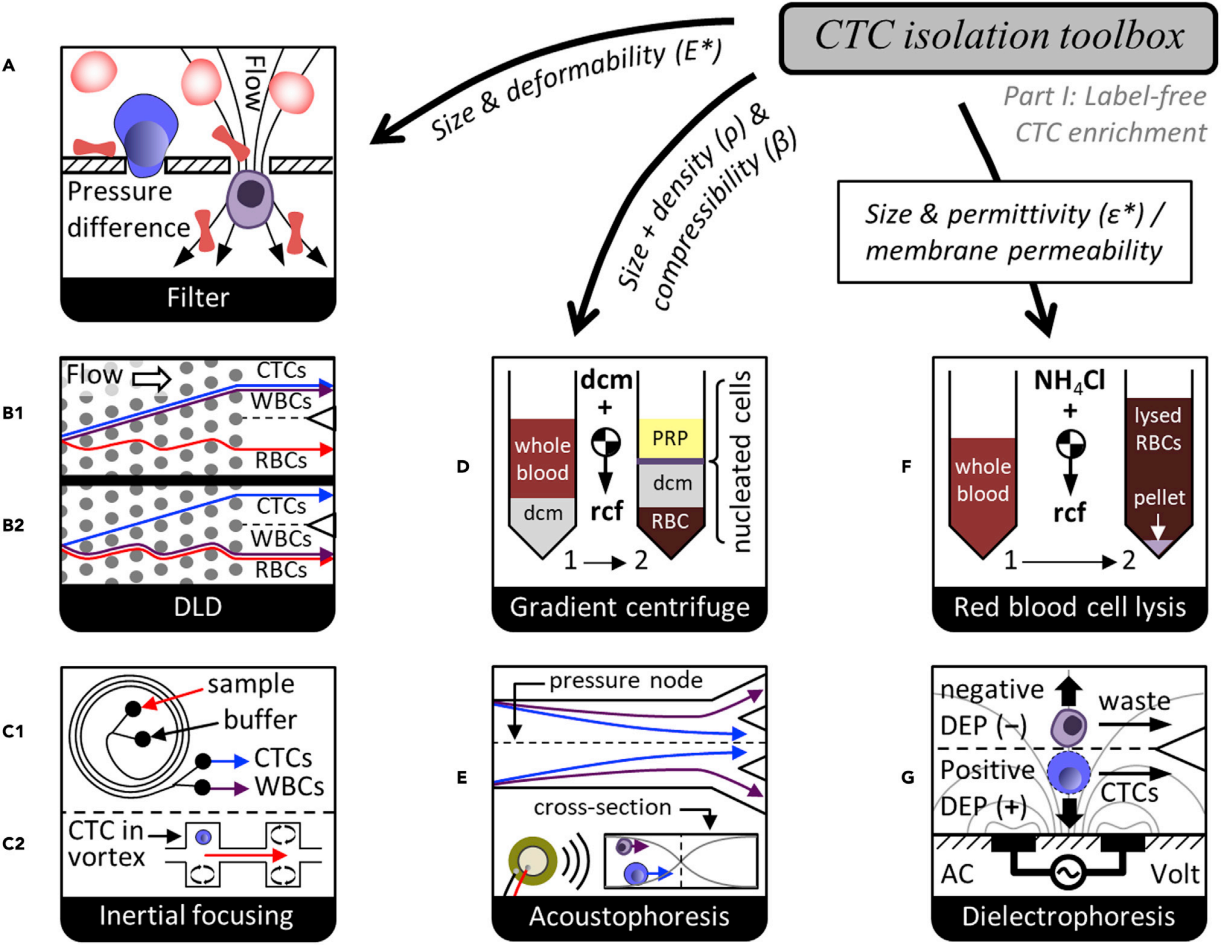
Label-free CTC enrichment Isolation strategies are constructed along multiple dimensions of separation for each population of normal blood cells and all or a subset of CTCs. This necessarily includes removing RBCs and platelets by size or other physical properties. Label-free isolation either accepts loss of CTCs with sizes or biophysical properties that overlap with WBCs, or leaves CTCs mixed with WBCs, relying purely on the above methods.

Essentially all CTC detection strategies require first that nucleated cells are cleared from the ubiquitous RBCs and platelets (debulking). One simple approach to debulking blood is centrifugation, whereby a “buffy-coat” layer, enriched for nucleated cells, accumulates on top of the packed RBC fraction. Separation can be improved by layering density centrifugation medium (dcm) beneath the blood prior to the spin (Figure 2D). Alternatively, selective RBC lysis through osmotic imbalance (Figure 2F) is possible due to the characteristic membrane transport properties and higher surface to volume ratio of biconcave RBCs relative to nucleated WBCs. In either case, remaining CTCs coexist with up to 5–10 million leukocytes per mL of blood. It is then possible to identify these rare cells via immune fluorescence (IF) imaging, yet detection is not label-free. For example, the high-throughput imaging and scoring technology of *Epic Sciences* (Wendel et al., 2012; Werner et al., 2015) first enriches CTCs by lysing RBCs with ammonium chloride, then plates all nucleated cells on two microscope slides per mL blood (CTCs are enumerated after staining and hand-validated scoring). Nearly all CTCs should be retained on the slides, and imaging normal leukocytes along with any CTCs can provide a kind of in-sample control against staining or processing variances. It does add a burden of specificity in discounting non-CTCs, and culture of these fixed cells is not possible.

### Enrichment of CTCs by size

**Figure 3:**
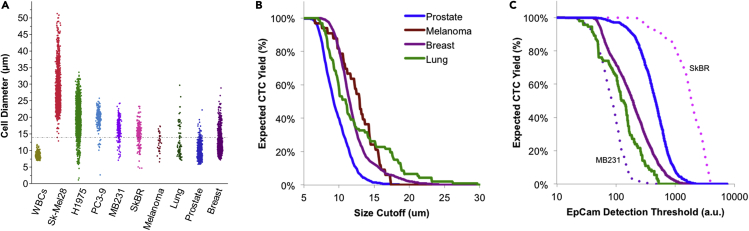
Predicted CTC yield with enrichment by size or positive selection We found as in (A) that patient-derived CTCs (melanoma, lung, prostate, and breast) are on average smaller than cultured cancer cells (from Sk-Mel28 to SkBR), overlapping with WBCs. Two waterfall plots predict CTC yield for (B) size- or (C) EpCAM-based isolation. Graphs from primary CTC-derived data are adapted without modification or warranty from Figures 2A and SI Figure 7 of (Fachin et al., 2017) under the *Creative Commons Attribution 4*.*0 International License*.

To further purify CTCs from nucleated cells, one premise is often applied: that most CTCs are larger than the leukocytes. While it is true that many cancer cell lines, CTCs, and clusters are larger than the average WBC, single CTCs can actually be quite small, even smaller than WBCs. For example, one study of 100 patients with prostate cancer revealed large numbers of very small CTCs with nuclei down to about 5 μm, and the presence of these cells correlated with visceral metastases (Chen et al., 2015). Toward the other end of scale, monocytes are about 12–20 μm in diameter (as viewed on blood smear) and number 0.5–1.3 million per mL blood (Rodak and Carr, 2013), overlapping the range of CTCs isolated by size (about 15 μm or more). As adapted in Figure 3A, we also found using size-independent CTC isolation that many patient-derived CTCs overlap WBCs in size (Fachin et al., 2017). Here, it is worth pointing out that primary CTCs (columns 7–10) were smaller on average than cultured cancer cells (columns 2–6). Based on these data, one can predict a progressive reduction in CTC yield for technologies relying on size sorting alone, as adapted in Figure 3B.

Filtration is perhaps the most direct form of size-sorting. For example, the *CellSieve* filter technology from Creatv MicroTech (Adams et al., 2014) captures CTCs by pushing blood through 160,000 cylindrical 7-μm pores. Leukocytes are too big to fit through based on size alone, so they must deform significantly to squeeze through (Figure 2A), enabling isolation of larger or stiffer CTCs. *Parsortix* also uses filtration to trap CTCs on-chip in a stepwise constriction which holds them for the duration of the isolation run (Miller et al., 2018). However, we demonstrated that CTC clusters may break into single cells while entering the accelerating flow near a constriction (Mutlu et al., 2020), risking loss of cluster-specific information. Additionally, CTC clusters can pass very narrow constrictions cell by cell if they deform over time (Au et al., 2016), though “seesaw” microstructures can prevent this single-file cluster transiting by isolating CTC clusters based on their non-spherical shape (Sarioglu et al., 2015). Moreover, filters struggle to sort by size alone given that cells are squishy.

Microfluidic technologies that utilize continuous-flow fractionation can in principle sort cells based on their undeformed shape. Deterministic lateral displacement (DLD) (Huang et al., 2004) is one key method of this type. DLD is built from a repeating array of microfluidic obstacles, between each of which a controlled fraction of flow is siphoned across the primary flow direction. Eventually, this flow shifting will bring every particle (or cell) into close contact with the obstacles, where, if the particle is large enough, the obstacle nudges it out of the siphoned streamlines. After successfully passing many such gaps, the larger particles will have migrated a significant distance across the flow, enabling their separation from smaller particles (which follow the siphoned flow). If a spherical particle remains in its undeformed shape, this can result in a sharp size cutoff for sorting.

But if a cell deforms during contact with an obstacle, it will delve deeper into the siphoned streamlines at each gap, broadening the size cutoff and shifting it to larger cells. Deformability is not a reliable dimension along which all CTCs may be separated from WBCs (Bagnall et al., 2015; Che et al., 2017). Nevertheless, flow-induced deformation helps a DLD to separate RBCs from nucleated cells (Economides et al., 2021) due to the absence/presence of the more rigid nucleus. In addition, though size cutoff is sharper when channel height far exceeds the width between obstacles, height-restricted DLDs can be used to sort clusters from single CTCs by their non-spherical shape (Au et al., 2017).

In practice, the requisite narrow channels and near cell-sized posts inherent to DLD are prone to manufacturing defects, and repeated collisions of cells with numerous posts can encourage clogging (Loutherback et al., 2012), yet DLD remains a commonly used method of microfluidic size sorting. As depicted in Figure 2B1, DLDs have been used to isolate CTCs and WBCs from blood, even depleting RBCs by over 10,000-fold, to deliver a clean nucleated cell fraction (Ozkumur et al., 2013). This buffy coat can be useful for high-throughput IF imaging without further purification, but for practically any other assay, there is a need to remove the WBCs. If one removes the WBCs by their size (Figure 2B2), many CTCs are lost, yet a high degree of specificity can be achieved by combining size sorting of CTCs from WBCs with IF imaging.

One size-based CTC sorter which does not require cells to contact obstacles or challenging fabrication is the *CTChip* technology. This approach uses inertial fluid forces (Di Carlo, 2009) in a spiral microfluidic chip (Khoo et al., 2014) to deflect large CTCs from lysed blood into a buffer co-flow for collection, as in Figure 2C1. Centrifugation then enables volume reduction for IF imaging to distinguish CTCs in the product from remaining WBCs. Alternatively as in Figure 2C2, micro-vortex traps have been developed that apply inertial lift forces to hold large CTCs in numerous vortex traps inside the chip (Dhar et al., 2015). This has the benefit of massive concentration of CTCs from blood to the on-chip traps, but cells are exposed to shear stress for full duration of the isolation process. To avoid high shear, array-based inertial focusing (Mutlu et al., 2017) was recently adapted to large 100-μm-wide channels to gently isolate intact CTC clusters from large blood volumes (Edd et al., 2020). Interestingly, during inertial focusing, more deformable cells shift further from the channel walls, effectively increasing their apparent size (Guzniczak et al., 2020; Martel and Toner, 2014). This further improves retention of less stiff CTCs and clusters.

### Separation by acoustic properties

In addition, acoustophoresis can separate cells based on physical properties. In acoustophoresis, piezoelectric transducers generate standing waves in a flowing fluid, leading to periodic forces on particles with non-zero time-averages. The acoustic radiation force is commonly used, and it depends on particle size, density, and compressibility (Petersson et al., 2007). Although cell size dominates for most acoustic cell separations, a size-independent acoustic focusing approach has been demonstrated (Augustsson et al., 2016). In this study, gradients of fluid acoustic impedance yield equilibrium focusing positions dependent on cell density and compressibility. Besides using external elements to create standing waves at channel resonant frequencies (Augustsson et al., 2012) as in Figure 2E, it is possible to create surface acoustic waves with inter-digitated electrode arrays placed atop a piezoelectric substrate in contact with the channel (Li et al., 2015). In most cases, vibrations are tuned so that the larger CTCs migrate to pressure nodes faster than WBCs, RBCs, and platelets, enabling dynamic separation.

### Separation by bioelectric properties

There can also be differences between the plasma membrane of CTCs and many of the leukocytes that manifest as altered bioelectric properties, and these can be applied for CTC enrichment from blood. For example, the *ApoStream* system from ApoCell (Gupta et al., 2012) uses an electrode array inside a microchannel to pull CTCs toward the electrode plane with positive dielectrophoresis (DEP) while pushing WBCs away from the electrodes into a separate waste stream by negative DEP, as depicted in Figure 2G. For DEP, separation arises because the crossover frequency at which cells switch from negative DEP to positive DEP under an AC applied electric field is lower for many CTCs (30–40 kHz) as compared to WBCs (90–140 kHz). Intracellular electrical conductivity and membrane composition play a role, but the shift in crossover frequency between CTCs and WBCs can be traced to the total capacitance of the cell membrane, proportional to membrane area, so separation benefits from some CTCs being larger or having a “rougher” membrane. Recently, the *DEPArray* system has emerged as a purification system for CTCs for secondary enrichment. It utilizes 30,000 DEP cages that can trap and levitate designated cells which can then be sorted and moved to retrieval chambers (Trapani et al., 2018).

The chief advantages of any label-free CTC isolation technology are that aside from the cost of running buffer or sample reagents (e.g. preservatives and fixatives), they can isolate larger CTCs at low cost, and CTC-WBC aggregates, such as circulating tumor-associated macrophages (Adams et al., 2014), are retained. However, to retain smaller CTCs, one needs to sort out the overlapping WBCs by other means.

## Enrichment by antigens specific to CTCs or WBCs

Up to now, we have discussed enrichment strategies that rely on geometric and biophysical differences between CTCs and other blood cells; however, these methods make broad assumptions about the nature of CTCs that can miss important biology, requiring tradeoffs between yield and purity if employed alone. Although CTCs are heterogeneous, they are non-hematological in origin (aside from blood malignancies) and many have distinctive biomolecules on their surfaces reflecting their tissue of origin. On the other hand, WBCs are well understood and express known antigens. Because highly specific antibodies to the key antigens on or in WBCs and some CTCs are available, and can be conjugated to fluorophores, surfaces, and magnetic beads, biomolecular differences between WBCs and CTCs have been turned toward CTC isolation technology.

### Positive selection by EpCAM: CTC-dependent isolation

**Figure 4:**
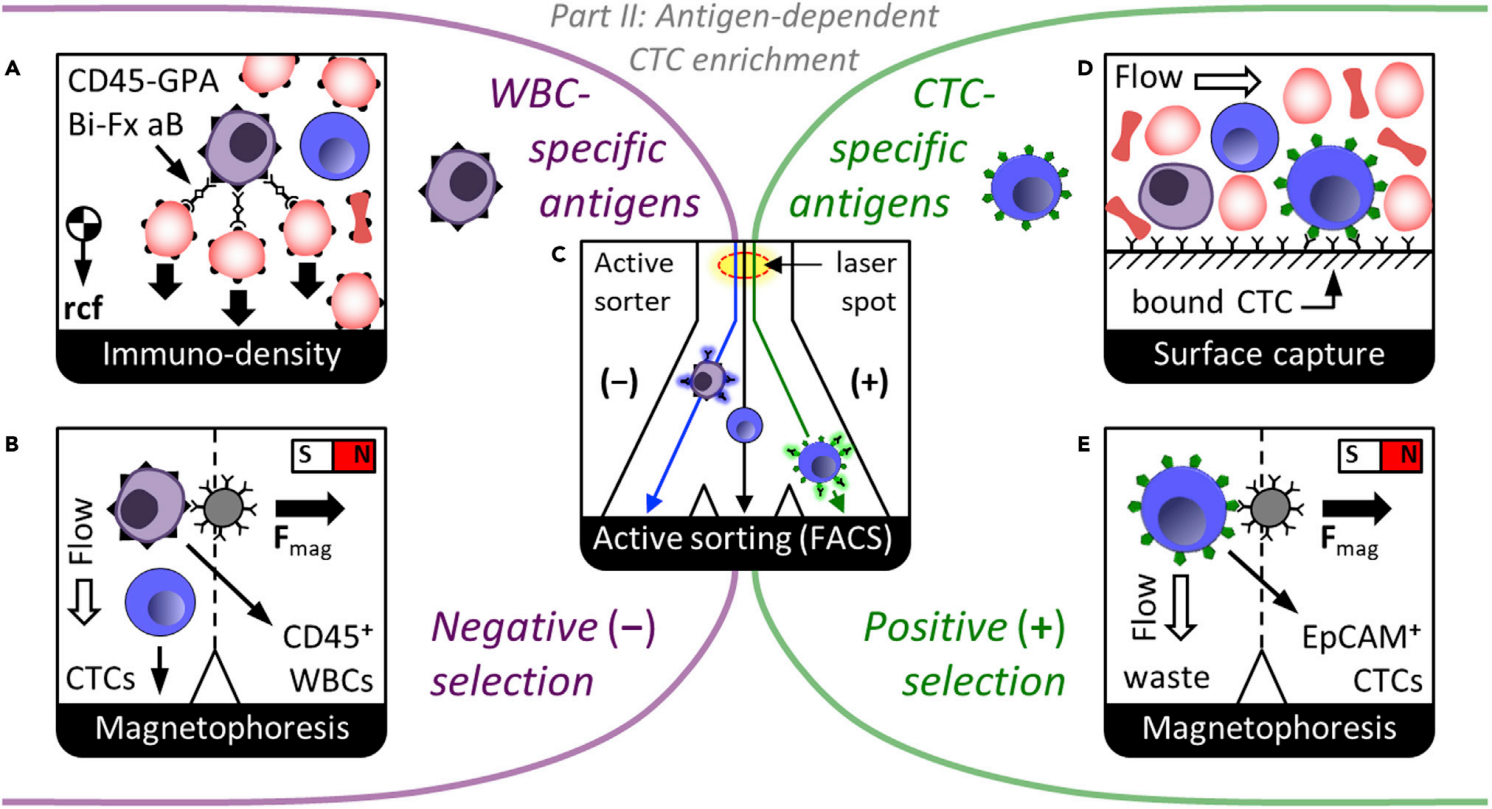
Antigen-dependent CTC enrichment To remove WBCs from CTCs, negative selection (AB) targets specific surface antigens (e.g. CD45). Alternately, positive selection (DE) purifies CTCs if they present surface-available EpCAM. (C) FACS can sort by multiple antigen-based or label-free metrics simultaneously.

A key advance in isolating CTCs irrespective of their physical properties has been the development of affinity-based capture of cells expressing EpCAM. Most solid cancers are epithelial in nature, and many CTCs retain expression of EpCAM on their plasma membranes after dissemination in the blood stream, while normal cells expressing EpCAM are very rare in the peripheral blood. Though EpCAM is often the most useful target, it is worth mentioning that other antigens have been successfully utilized for positive selection of CTCs in blood. These include PSMA to capture prostate CTCs (Gleghorn et al., 2010) and HER2 to capture breast cancer CTCs (Xu et al., 2011). Whether EpCAM or another antigen is targeted, the key advantage of affinity-based positive selection is that it isolates CTCs regardless of their size, shape, or aggregation with immune cells. Equally important is that high purity is possible by washing since EpCAM is a high-affinity CTC handle, even when sorting cells directly from whole blood (Nagrath et al., 2007; Powell et al., 2012). *CELLSEARCH*^*TM*^ (Janssen Diagnostics) (Cristofanilli et al., 2004), which is an FDA-approved technology for CTC isolation, labels CTCs with magnetic beads coated with antibodies to EpCAM and then isolates them by magnetic-activated cell sorting (MACS, as in Figure 4E). IF imaging then enumerates CTCs as nucleated cells positive for cytokeratin 8, 18, or 19 and negative for CD45, formerly known as the leukocyte common antigen. This MACS approach was later joined by the *positive* selection microfluidic *CTC−iChip* technology (Ozkumur et al., 2013) with improved sensitivity for low-EpCAM CTCs. This microfluidic method uses DLDs to remove RBCs, platelets, plasma, and unbound magnetic beads based primarily on size. Next, it focuses all nucleated cells into a single file with inertial focusing channels prior to high-gradient microfluidic MACS to pull EpCAM-positive CTCs to the product. A key advance is a reduction in required lateral distance for magnetic separation afforded by inertial focusing: cells need not travel far to enter the product. Moreover, although positive MACS can find very small CTCs with ease, it is dependent on EpCAM, and beads remain bound to CTCs.

Besides MACS, anti-EpCAM antibodies have also been employed for surface-based capture (Figure 4D) of CTCs using microfluidics. These affinity-based methods do not require beads and enable real-time visualization of CTCs. The relatively small capture areas involved require non-specific binding and fouling to be overcome by special coatings and carefully designed flow profiles to maximize specific CTC capture at high blood throughput. Examples include coated post arrays (Gleghorn et al., 2010; Nagrath et al., 2007) and passive herringbone mixing channels (Stott et al., 2010b).

### Negative selection to remove WBCs: CTC-independent isolation

EpCAM-based positive selection can miss important biology, including, for example CTCs from non-epithelial cancers such as melanoma and sarcoma, CTCs that have undergone an epithelial-mesenchymal (EMT) transition, or platelet-coated “stealth” CTCs. This key weakness of positive selection is illustrated in Figure 3C, where CTC yield is plotted against the threshold of EpCAM expression on the membrane (Fachin et al., 2017). Moreover, the field of CTC biology is evolving and CTCs themselves are diverse across cancer type, disease stage, treatment regimen, and in a single blood draw because of the likelihood of CTCs arising from multiple clonal populations and separate tumors.

One approach to isolate all CTC populations with high purity regardless of EpCAM or physical properties is to focus on removing the WBCs based on their biochemical traits not shared by CTCs. This can be accomplished based on the presence of well-known and ubiquitous cell surface antigens. Fortunately, nature provides an ideal target, CD45, which is present on >99% of WBCs in most samples from healthy donors or patients and can be used in negative MACS (Figure 4B). This approach has been applied in a mini-fluidic continuous flow-through system (Lara et al., 2004), using RBC lysis as a first step, to achieve a high degree of WBC removal. In addition, the *negative CTC-iChip* technology (Ozkumur et al., 2013) which added CD16 and CD66b, achieved removal of 99.99% of leukocytes after debulking by DLD and two stages of inertial focusing and microfluidic MACS in a continuous flow. This original *CTC-iChip* used DLDs to separate WBCs and CTCs from smaller RBCs, platelets, plasma, and unbound beads (Fachin et al., 2017; Ozkumur et al., 2013). Next, a purified stream of nucleated cells is aligned passively by fluid forces (Di Carlo et al., 2007; Martel et al., 2015) for microfluidic negative selection (Fachin et al., 2017; Karabacak et al., 2014; Ozkumur et al., 2013), removing bead-labeled WBCs from untouched CTCs, regardless of EpCAM expression. Here, inertial focusing is beneficial in preventing collateral loss of CTCs as the many millions of WBCs are swept out of the nucleated cell stream.

**Figure 5:**
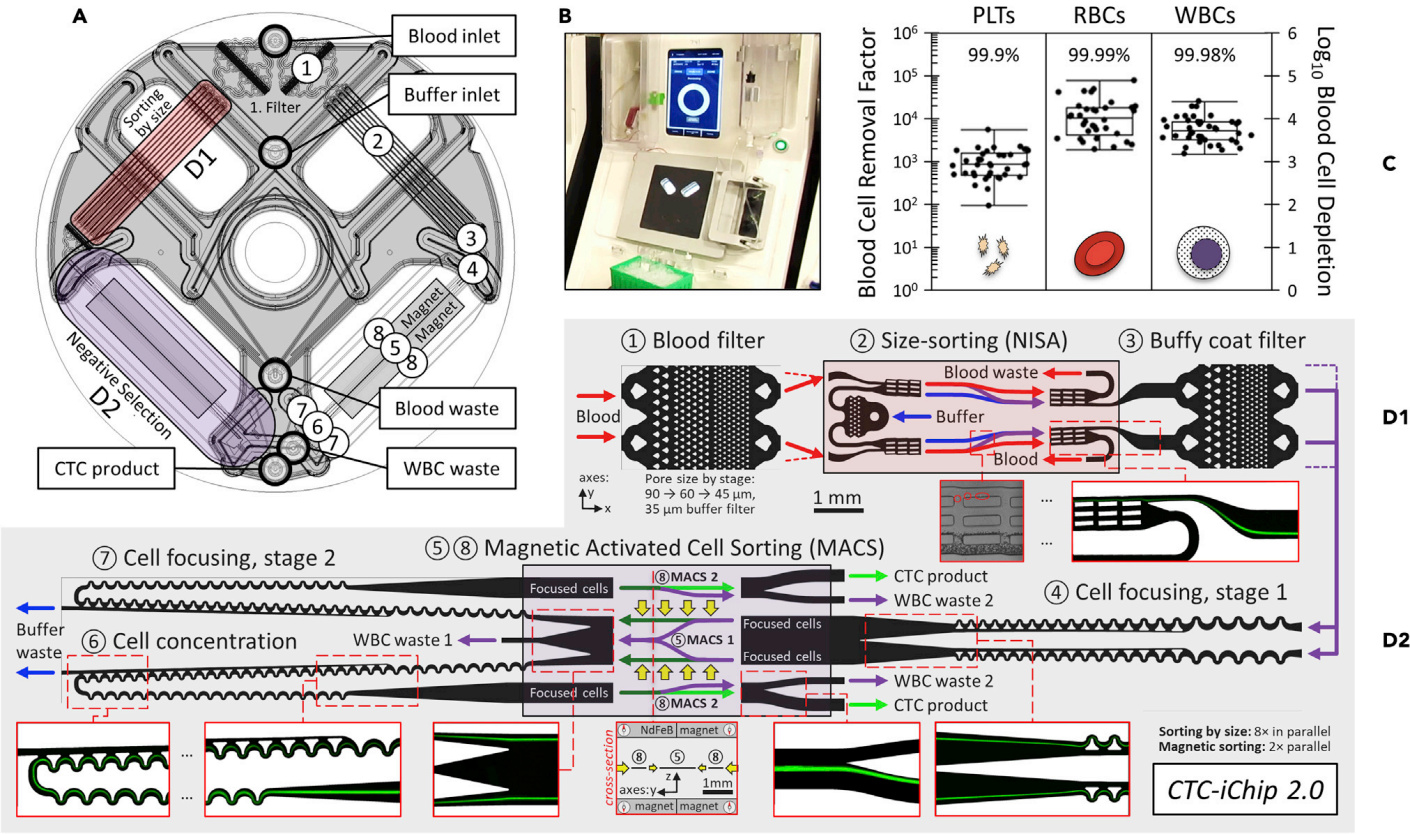
*CTC-iChip 2* As depicted in (A), *CTC-iChip 2* combines two modules: size sorting to separate nucleated cells from blood and negative selection to remove bead-labeled WBCs from untouched CTCs. Shaded area is laser-welded macro-micro interface that connects injection-molded microchannel devices (52 μm tall) to 5 tubing ports labeled in (A) and visible in image of chip in processor (B). *CTC-iChip 2* has bilateral symmetry, and each numbered stage is detailed in (D1) for size sorting and in (D2) for negative selection, from inlet to outlet. Inset images trace the path of untouched nucleated cells at key locations. After a final stage of magnetic-activated cell sorting, CTCs exit the chip, enriched for downstream analysis as tabulated in (C) for platelets, RBCs, and WBCs (whiskers span the full range of data; box encloses the interquartile range and the 50^th^ percentile line). Size sorting is as in (Mutlu et al., 2017). Magnets are arranged as in (Fachin et al., 2017). Blood samples were collected from healthy volunteers following experimental protocols reviewed and approved by the Massachusetts General Hospital IRB, where protocol numbers 2009-P-000295 and 2015-P-000656 were used to obtain informed consent from internal donors and donors at the MGH Blood Bank, respectively. Some healthy samples were also ordered from Research Blood Components, LLC (Brighton, MA). All samples were obtained in accordance with the applicable federal guidelines and regulations.

Though *CTC-iChip* could process 10 mL of blood per hour, the very narrow microchannels of its 128 parallel DLD arrays limited scalability to larger blood volumes and incurred loss of CTCs as small as the lower half of WBCs. DLDs also suffer from shear-induced clogging (Loutherback et al., 2012; Wong et al., 2018) made worse by prolonged contact of blood with tiny posts in narrow channels. Since the original DLD-based *CTC-iChip*, blood throughput has been raised by replacing the DLD debulking modules with an array-format inertial focusing device, as described in (Mutlu et al., 2017). This removed hundreds of pointwise collisions between CTCs and DLD posts in favor of separating nucleated cells from the blood with a continuous inertial lift force, resulting in greater reliability and throughput. To meet a higher blood throughput upstream, MACS was also accelerated by incorporation of cell concentrators built from inertial focusing (Martel et al., 2015), reducing NISA product flow rate 8.5× by the time CTCs enter MACS stage 2 (Fachin et al., 2017), thereby retaining high end-to-end WBC depletion. As originally reported here in Figure 5, the updated architecture of *CTC-iChip 2* speeds blood debulking by 10-fold per array, while simultaneously lowering the size cutoff, to isolate CTCs that overlap in size with even the smallest WBCs. The modular design is shown in Figure 5D, blood cell depletion performance is given in Figure 5C, and its companion instrument is depicted in Figure 5B.

Considering other approaches to negative selection, the *RosetteSep* technology (STEMCELL Technologies) includes a reagent kit for CTC isolation by immunodensity removal of WBCs (Figure 4A). It uses tetrameric antibody complexes to bind RBCs to leukocytes for density gradient centrifugation. RBC-bound WBCs become denser and sediment to the RBC fraction, while unbound CTCs remain in the product. Finally, other microfluidic approaches to negative selection are emerging, including NiFe-coated track-etched filters to provide parallel magnetic trapping of WBCs (Ko et al., 2017).

In summary, negative selection has the advantage over positive selection of leaving CTCs untouched, and any required size cutoff for isolation may be set just above the RBCs, so small CTCs are retained unless they are extremely small. Non-specific binding endangers yield in negative selection, so reagents must be optimized to avoid CTC loss. Clusters of CTCs and WBCs would be lost if they are not first removed by size sorting (Edd et al., 2020).

### Fluorescence-activated cell sorting (FACS)

The isolation technologies mentioned above all intrinsically unify the steps of sensing and actuation so that large numbers of cells can be sorted at high speed. For example, MACS “senses” cells by bead binding via antigen-antibody binding and “actuates” all at once with an applied magnetic field. Approaches based on biophysical properties (e.g. size, density, deformability, or dielectric properties) combine sensing and actuation even more intimately. On the other hand, active cell sorting, as in commercial jet-in-air FACS systems, separates these actions so the decision to sort, and into which fraction, is based on a set of logical gates. The advantages for CTC isolation from blood are clear: 1) negative or positive selection can be based on light scattering characteristics and as many as eighteen high dynamic range measurements of fluorescent-labeled markers, 2) the profile of each cell is recorded for later study, and 3) the sample can be subdivided into several product streams for later analysis. For example, the *FACSAria III* system from BD claims to sort cells at >98% purity and >80% of *Poisson’s* expected yield at 25,000 cells per second. This system could sort the ∼60 million WBCs in a 7.5 mL tube of blood in about 40 min, although removing RBCs and platelets is a prerequisite. Moreover, high-speed flow focusing followed by acoustic droplet-in-air formation and impact in a collection vial puts potentially apoptotic CTCs or fragile clusters at risk of damage. These systems are also expensive, requiring multiple lasers, sophisticated electronics, and dedicated help.

To reduce cost and handle cells more gently, and in a closed system to avoid cross-contamination, disposable microfluidic FACS can be adapted for rare cells (Figure 4C). For example, the *eDAR* system (Zhao et al., 2013) sorts packets of blood with CTCs based on pulses of light emanating from fluorescently labeled EpCAM-positive cells. RBCs, platelets, and plasma are then removed from nucleated cells by a downstream size-based filter. Because CTCs are rare and the packet of blood which is hydrodynamically switched to the filter is small, WBC carryover is low as in other positive selection methods. In addition, Sony Biotechnology markets the SH800 microfluidic FACS system, and Cytonome recently introduced *GigaSort*, *using* flow switching. It can sort up to 48,000 cells a second with a disposable microfluidic cartridge and 24 parallel sorters and would be able to sort CTCs from debulked nucleated cells by FACS. Nevertheless, FACS seems less likely to be used for primary sorting of CTCs from large blood volumes. These flow sorting systems, as well as image-based cell picking (Donato et al., 2019), can be well adapted for secondary sorting of the CTC-enriched product, owing to typically 1000-fold or more reduced numbers of input contaminating cells.

## Large volume

Standard phlebotomy most often provides 1 to 2 vacutainers of blood from a patient with cancer, in which on the order of 10 CTCs could be present. Considering this extreme rarity along with the minimum number of CTCs required for many downstream assays, there have been various approaches taken to increase the analyzed blood volume, to increase overall assay sensitivity. This is an absolute necessity for early detection, or when CTCs are present at frequencies <1 CTC/10 mL peripheral blood. For example, Nagrath and colleagues proposed an intravascular apheretic system where blood is processed through anti-EpCAM-coated herringbone channels for continuous positive selection of CTCs (Kim et al., 2019). An indwelling functionalized medical wire for *in vivo* collection of CTCs from the blood has also been proposed (Saucedo-Zeni et al., 2012); however, it captures cells from a small area near the wire, reducing effective processed volume. An intravascular magnetic wire for *in vivo* immunomagnetic capture of CTCs (Vermesh et al., 2018) has also been outlined, requiring *in vivo* labeling of cells with magnetic particles, though about 4% of injected magnetic particles were not cleared by the magnetic wire. Moreover, these approaches rely on cell surface markers such as EpCAM and may miss the CTCs that exhibit lower or negligible EpCAM expression (Figure 3C) due, for example, to dynamic epithelial-mesenchymal transition.

More recently, there has been an increasing interest in using leukapheresis to obtain large volumes of concentrated WBCs and CTCs (Eifler et al., 2011; Fischer et al., 2013; Franken et al., 2019; Lambros et al., 2018). Leukapheresis products are obtained by automated centrifugal enrichment of 1 to 3 L of peripheral blood, and they could represent ideal samples for enhancing CTC yields 50- to 100-fold over a 10 mL blood sample (Andree et al., 2018). See Figure S1 from (Mishra et al., 2020) for a mathematical model of the benefits to sampling statistics brought by leukapheresis over phlebotomy. Leukapheresis is a standard and well-tolerated clinical procedure in apheresis centers. In this process, peripheral blood cells are centrifugally collected into different layers based on their density, such that mononuclear cells can be siphoned into a Leukopak (Fischer et al., 2013; Lambros et al., 2018), while the remaining components of the blood (RBCs, platelets, and neutrophils) are continuously returned to the patient. CTCs often have a similar density as mononuclear cells (1050–1080 kg/m^3^), and as a result they become enriched in the leukapheresis product (Fischer et al., 2013).

Yet this sample remains challenging for CTC isolation due to the presence of billions of WBCs. Using positive selection (CELLSEARCH), Lambros et al. isolated CTCs from whole blood and a small fraction of leukapheresis products for 14 patients with prostate cancer. They reported an increase from 167 CTCs found in the 7.5 mL blood samples to 1,918 CTCs from just 5% of the leukapheresis product. Fehm et al. isolated up to ∼2,150 CTCs by again processing just 5% of leukapheresis products from 40 patients with breast cancer (Fehm et al., 2018). Fischer et al. analyzed 29 leukapheresis samples from breast, pancreatic, colorectal, and esophageal cancers and found CTCs in 21 out of 29 samples whereas only 8 out of 29 peripheral blood samples contained CTCs (Fischer et al., 2013). These studies are consistent with the expectation that 100-fold more CTCs could be collected from the blood by leukapheresis.

However, positive selection CTC isolation technologies developed for whole blood mostly process up to 200 million mononuclear cells (3%–5% of a leukapheresis product) (Andree et al., 2018; Fehm et al., 2018; Fischer et al., 2013; Franken et al., 2019; Lambros et al., 2018; Reinhardt et al., 2019). This is primarily due to the characteristics of a leukapheresis product: (i) a high concentration and 100-fold larger number of nucleated cells relative to a standard tube of blood, (ii) large and highly variable numbers of RBCs and platelets depending on leukapheresis parameters, and (iii) a large fluid volume (60 to 120 mL). These traits make isolating CTCs from a leukapheresis product incompatible with almost all existing microfluidic approaches. In particular, magnetic sorting of WBCs from leukapheresis product is difficult due to the huge number of bead-labeled WBCs that can create clogging aggregates in bulk and in microfluidic sorting devices.

Mishra et al. recently addressed some of these challenges by creating a permeability-enhanced magnetic sorter that uses high-magnetic-permeability material (soft-iron) adjacent to high-speed sorting channels. This arrangement enhances the magnetic field gradient and increases cell sorting throughput (Mishra et al., 2020). The high-permeability channels act as on-chip magnetic micro-lenses and enhance the magnetic force 35-fold over the *CTC-iChip* design in the sorting channels (Figure 5, part D2). Using this magnetic lensing approach, Mishra et al. showed processing of 6 billion WBCs from leukapheresis products in an hour using two devices at a throughput of 168 mL/h, with 86.1 ± 0.6% CTC recovery and 99.97% depletion of WBCs in negative selection mode. Blood cell removal by size was by *NISA* (Mutlu et al., 2017).

This enables processing of the full leukapheresis product while recovering even EpCAM-negative CTCs. Yet to ensure clusters of CTCs and WBCs are also retained, and CTC clusters remain intact, we also recently reported a gentle high-throughput device for CTC cluster sorting from blood (Edd et al., 2020). When applied before negative selection, potential loss of CTC-WBC aggregates or breakup of CTC clusters can be avoided, yielding a combined strategy to sort all CTC types from a full leukapheresis product.

## Outlook

Tools for cell biology have advanced quite rapidly in the last few years, in the precipitously falling costs of next-gen sequencing, molecular bar-coding, commercialization of high-throughput digital PCR, and advances in proteomics. Besides accelerating the pace of decoding the molecular biology of cancer, and by extension CTCs, these trends may shift focus away from strict CTC enumeration and toward genotyping, expression analysis, and single-cell sequencing for genomics and transcriptomics (Zheng et al., 2017). Nevertheless, FDA approval was granted for one CTC enumeration platform already (CELLSEARCH), and since CTC numbers were correlated to patient outcomes (Cristofanilli et al., 2004), IF-based enumeration is likely to remain a part of many CTC detection strategies in the near to mid-term. However, IF imaging has significant bandwidth limitations and often struggles to detect multiple biomarkers with sufficient specificity, making high CTC purity a key requirement.

Advancing assay technologies may loosen constraints on CTC isolation technologies, including acceptable levels of WBCs and other impurities, allowing isolation to broaden the net for catching CTCs with overlapping features to normal blood cells. Notably, digital PCR has improved multiplexing of biomarkers and retains a higher specificity despite the presence of many more blood cells. Together, this allows detection of fewer CTCs among more biomatter, all at lower cost and higher sample throughput. For example, we applied digital RNA-PCR to simultaneously detect tumor burden and lineage-specific CTC signaling pathways from *CTC-iChip*-enriched patient blood samples in melanoma (Hong et al., 2018) and cancers of the breast (Kwan et al., 2018), prostate (Miyamoto et al., 2018), and liver (Kalinich et al., 2017). Considering the rarity of CTCs in blood, and the reduced purity requirements of assays like digital PCR, isolation technology should prioritize high yield of all CTC types in the largest relevant blood volumes. Purity of the product could be of secondary importance, provided it is sufficient for the downstream assays. In a context of an ever-increasing blood volume for isolation, FACS may lack the needed throughput on its own. In addition, size-based isolation and positive selection miss many CTC types (Figure 3), yet isolation by size can be the simplest approach to scale.

In contrast, combining inclusive size sorting with negative selection provides the broadest isolation of all CTC types in large blood volumes and will have a higher yield than bulk separation due to precise cell handling of microfluidics. Applied to leukapheresis products, negative selection can isolate 100-fold more CTCs, enhancing reliability and sensitivity of CTC-based assays. It will also increase the success rate of ex-vivo culture and help open the window into CTC heterogeneity. Yet there is room for improvement since CTC-WBC clusters could be lost, suggesting an upstream size/shape-based isolation module. The resulting integrated isolation technology would retain a high yield for every CTC larger than an RBC with the possibility to achieve apheresis of liters of blood.

Besides CTC-based assays, there is currently a great deal of focus being given to commercial and academic research to detect mutations within ctDNA (Cescon et al., 2020) and also to tumor-related gene expression via cell-free RNA and that held within tumor-shed exosomes (Yu et al., 2021) or micro-vesicles. Rather than being mutually exclusive, a convincing argument can be made for combining the readouts of multiple blood-based biomarkers to raise both sensitivity and specificity for early detection and better integrate to treatment decisions. Notably, ctDNA technology has undergone a rapid expansion, especially in disease monitoring and identifying therapeutically actionable tumor alterations (Ignatiadis et al., 2021). Yet still a low amount of ctDNA in patients at early stage may make cancer screening challenging (Cescon et al., 2020), with *Galleri* achieving an overall sensitivity of 16.8% in stage 1 cancers (Klein et al., 2021). A synergistic potential was also demonstrated in combining mutation detection from ctDNA with detection of tumor-modulated proteins (Cohen et al., 2018) to increase overall assay sensitivity.

CTCs have the unique characteristic within liquid biopsy that DNA, RNA, and protein are all colocalized in a single cell, so in theory they should provide exquisite specificity. Even if molecular techniques sometimes struggle to elucidate these details within every isolated CTC among a much larger population of unwanted cells, CTC genotyping can still provide the mutation profile of multiple clonal populations with high confidence, enabling correlation with ctDNA sequencing to piece together the mutated sequences. Single-cell RNAseq may be emerging as the assay of choice for tracing the response to treatment (gene expression), and even drug testing for live CTC isolates. Looking forward, the breadth and depth of our CTC knowledge will improve along with CTC-independent isolation technologies and the greater quantity and quality of information extracted from each CTC. This in its turn will continue to spur further advancements in isolation technology as previously unknown aspects of CTC biology become understood and their clinical utility becomes clear.

This comes to a key point; utility for these highly specific CTC-details must be established in clinical trials to the satisfaction of the relevant regulatory bodies if they will be used in treatment decisions, and the rich information that can be obtained from even a single CTC should ideally be synthesized into higher level, actionable information to speed interpretation by clinicians. Only by converting isolated CTCs into actionable information can the potential for CTC isolation technology in oncology be realized.

## References

[bib1] Aceto N., Bardia A., Miyamoto D.T., Donaldson M.C., Wittner B.S., Spencer J.A., Yu M., Pely A., Engstrom A., Zhu H. (2014). Circulating tumor cell clusters are oligoclonal precursors of breast cancer metastasis. Cell.

[bib2] Aceto N., Toner M., Maheswaran S., Haber D.A. (2015). En route to metastasis: circulating tumor cell clusters and epithelial-to-mesenchymal transition. Trends in Cancer.

[bib3] Adams D.L., Martin S.S., Alpaugh R.K., Charpentier M., Tsai S., Bergan R.C., Ogden I.M., Catalona W., Chumsri S., Tang C.-M. (2014). Circulating giant macrophages as a potential biomarker of solid tumors. Proc. Natl. Acad. Sci. USA.

[bib4] Andree K.C., Mentink A., Zeune L.L., Terstappen L.W.M.M., Stoecklein N.H., Neves R.P., Driemel C., Lampignano R., Yang L., Neubauer H. (2018). Toward a real liquid biopsy in metastatic breast and prostate cancer: diagnostic LeukApheresis increases CTC yields in a European prospective multicenter study (CTCTrap). Int. J. Cancer.

[bib5] Ashworth T.R. (1869). A case of cancer in which cells similar to those in the tumours were seen in the blood after death. Med. J. Aust..

[bib6] Au S.H., Edd J.F., Stoddard A.E., Wong K.H.K., Fachin F., Maheswaran S., Haber D.A., Stott S.L., Kapur R., Toner M. (2017). Microfluidic isolation of circulating tumor cell clusters by size and asymmetry. Sci. Rep..

[bib7] Au S.H., Storey B.D., Moore J.C., Tang Q., Chen Y.L., Javaid S., Sarioglu A.F., Sullivan R., Madden M.W., O’Keefe R. (2016). Clusters of circulating tumor cells traverse capillary-sized vessels. Proc. Natl. Acad. Sci. USA.

[bib8] Augustsson P., Karlsen J.T., Su H.W., Bruus H., Voldman J. (2016). Iso-acoustic focusing of cells for size-insensitive acousto-mechanical phenotyping. Nat. Commun..

[bib9] Augustsson P., Magnusson C., Nordin M., Lilja H., Laurell T. (2012). Microfluidic, label-free enrichment of prostate cancer cells in blood based on acoustophoresis. Anal. Chem..

[bib10] Bagnall J.S., Byun S., Begum S., Miyamoto D.T., Hecht V.C., Maheswaran S., Stott S.L., Toner M., Hynes R.O., Manalis S.R. (2015). Deformability of tumor cells versus blood cells. Sci. Rep..

[bib11] Barteneva N.S., Ketman K., Fasler-Kan E., Potashnikova D., Vorobjev I.A. (2013). Cell sorting in cancer research--diminishing degree of cell heterogeneity. Biochim. Biophys. Acta.

[bib12] Cescon D.W., Bratman S.V., Chan S.M., Siu L.L. (2020). Circulating tumor DNA and liquid biopsy in oncology. Nat. Cancer.

[bib13] Che J., Yu V., Garon E.B., Goldman J.W., Di Carlo D. (2017). Biophysical isolation and identification of circulating tumor cells. Lab Chip.

[bib14] Chen J.F., Ho H., Lichterman J., Lu Y.T., Zhang Y., Garcia M.A., Chen S.F., Liang A.J., Hodara E., Zhau H.E. (2015). Subclassification of prostate cancer circulating tumor cells by nuclear size reveals very small nuclear circulating tumor cells in patients with visceral metastases. Cancer.

[bib15] Cho H., Kim J., Song H., Sohn K.Y., Jeon M., Han K.-H. (2018). Microfluidic technologies for circulating tumor cell isolation. Analyst.

[bib16] Cohen J.D., Li L., Wang Y., Thoburn C., Afsari B., Douville C., Javed A.A., Wong F., Mattox A., Hruban R.H. (2018). Detection and localization of surgically resectable cancers with a multi-analyte blood test. Science.

[bib17] Coumans F.A.W., Siesling S., Terstappen L.W.M.M. (2013). Detection of cancer before distant metastasis. BMC Cancer.

[bib18] Cristofanilli M., Budd G.T., Ellis M.J., Stopeck A., Matera J., Miller M.C., Reuben J.M., Doyle G.V., Allard W.J., Terstappen L.W. (2004). Circulating tumor cells, disease progression, and survival in metastatic breast cancer. N. Engl. J. Med..

[bib19] Dhar M., Wong J., Karimi A., Che J., Renier C., Matsumoto M., Triboulet M., Garon E.B., Goldman J.W., Rettig M.B. (2015). High efficiency vortex trapping of circulating tumor cells. Biomicrofluidics.

[bib20] Di Carlo D. (2009). Inertial microfluidics. Lab. Chip..

[bib21] Di Carlo D., Irimia D., Tompkins R.G., Toner M. (2007). Continuous inertial focusing, ordering, and separation of particles in microchannels. Proc. Natl. Acad. Sci. USA.

[bib22] Donato C., Szczerba B.M., Scheidmann M.C., Castro-Giner F., Aceto N. (2019). Micromanipulation of circulating tumor cells for downstream molecular analysis and metastatic potential assessment. J. Vis. Exp..

[bib23] Economides A., Arampatzis G., Alexeev D., Litvinov S., Amoudruz L., Kulakova L., Papadimitriou C., Koumoutsakos P. (2021). Hierarchical Bayesian uncertainty quantification for a model of the red blood cell. Phys. Rev. Appl..

[bib24] Edd J.F., Mishra A., Dubash T.D., Herrera S., Mohammad R., Williams E.K., Hong X., Mutlu B.R., Walsh J.R., Machado De Carvalho F. (2020). Microfluidic concentration and separation of circulating tumor cell clusters from large blood volumes. Lab. Chip..

[bib25] Eifler R.L., Lind J., Falkenhagen D., Weber V., Fischer M.B., Zeillinger R. (2011). Enrichment of circulating tumor cells from a large blood volume using leukapheresis and elutriation: proof of concept. Cytom. Part B Clin. Cytom..

[bib26] Engell H.C. (1955). Cancer cells in the circulating blood; a clinical study on the occurrence of cancer cells in the peripheral blood and in venous blood draining the tumour area at operation. Acta Chir. Scand. Suppl..

[bib27] Fachin F., Spuhler P., Martel-Foley J.M., Edd J.F., Barber T.A., Walsh J., Karabacak M., Pai V., Yu M., Smith K. (2017). Monolithic chip for high-throughput blood cell depletion to sort rare circulating tumor cells. Sci. Rep..

[bib28] Fehm T.N., Meier-Stiegen F., Driemel C., Jäger B., Reinhardt F., Naskou J., Franken A., Neubauer H., Neves R.P.L., van Dalum G. (2018). Diagnostic leukapheresis for CTC analysis in breast cancer patients: CTC frequency, clinical experiences and recommendations for standardized reporting. Cytometry.

[bib29] Fischer J.C., Niederacher D., Topp S.A., Honisch E., Schumacher S., Schmitz N., Zacarias Föhrding L., Vay C., Hoffmann I., Kasprowicz N.S. (2013). Diagnostic leukapheresis enables reliable detection of circulating tumor cells of nonmetastatic cancer patients. Proc. Natl. Acad. Sci. USA.

[bib30] Franken A., Driemel C., Behrens B., Meier-Stiegen F., Endris V., Stenzinger A., Niederacher D., Fischer J.C., Stoecklein N.H., Ruckhaeberle E. (2019). Label-free enrichment and molecular characterization of viable circulating tumor cells from diagnostic leukapheresis products. Clin. Chem..

[bib31] Gleghorn J.P., Pratt E.D., Denning D., Liu H., Bander N.H., Tagawa S.T., Nanus D.M., Giannakakou P.A., Kirby B.J. (2010). Capture of circulating tumor cells from whole blood of prostate cancer patients using geometrically enhanced differential immunocapture (GEDI) and a prostate-specific antibody. Lab. Chip..

[bib32] Gupta V., Jafferji I., Garza M., Melnikova V.O., Hasegawa D.K., Pethig R., Davis D.W. (2012). ApoStream^TM^, a new dielectrophoretic device for antibody independent isolation and recovery of viable cancer cells from blood. Biomicrofluidics.

[bib33] Guzniczak E., Otto O., Whyte G., Willoughby N., Jimenez M., Bridle H. (2020). Deformability-induced lift force in spiral microchannels for cell separation. Lab. Chip..

[bib34] Haber D.A., Velculescu V.E. (2014). Blood-based analyses of cancer: circulating tumor cells and circulating tumor DNA. Cancer Discov..

[bib35] Hamza B., Miller A.B., Meier L., Stockslager M., Ng S.R., King E.M., Lin L., DeGouveia K.L., Mulugeta N., Calistri N.L. (2021). Measuring kinetics and metastatic propensity of CTCs by blood exchange between mice. Nat. Commun..

[bib36] Hamza B., Ng S.R., Prakadan S.M., Delgado F.F., Chin C.R., King E.M., Yang L.F., Davidson S.M., DeGouveia K.L., Cermak N. (2019). Optofluidic real-time cell sorter for longitudinal CTC studies in mouse models of cancer. Proc. Natl. Acad. Sci. USA.

[bib37] Hanahan D., Weinberg R.A. (2011). Hallmarks of cancer: the next generation. Cell.

[bib38] Hong X., Sullivan R.J., Kalinich M., Kwan T.T., Giobbie-Hurder A., Pan S., LiCausi J.A., Milner J.D., Nieman L.T., Wittner B.S. (2018). Molecular signatures of circulating melanoma cells for monitoring early response to immune checkpoint therapy. Proc. Natl. Acad. Sci. USA.

[bib39] Huang L.R., Cox E.C., Austin R.H., Sturm J.C. (2004). Continuous particle separation through deterministic lateral displacement. Science.

[bib40] Ignatiadis M., Sledge G.W., Jeffrey S.S. (2021). Liquid biopsy enters the clinic — implementation issues and future challenges. Nat. Rev. Clin. Oncol..

[bib41] Ilie M., Hofman V., Long-Mira E., Selva E., Vignaud J.-M., Padovani B., Mouroux J., Marquette C.-H., Hofman P. (2014). “Sentinel” circulating tumor cells allow early diagnosis of lung cancer in patients with chronic obstructive pulmonary disease. PLoS One.

[bib42] Jordan N.V., Bardia A., Wittner B.S., Benes C., Ligorio M., Zheng Y., Yu M., Sundaresan T.K., Licausi J.A., Desai R. (2016). HER2 expression identifies dynamic functional states within circulating breast cancer cells. Nature.

[bib43] Kalinich M., Bhan I., Kwan T.T., Miyamoto D.T., Javaid S., LiCausi J.A., Milner J.D., Hong X., Goyal L., Sil S. (2017). An RNA-based signature enables high specificity detection of circulating tumor cells in hepatocellular carcinoma. Proc. Natl. Acad. Sci. USA.

[bib44] Karabacak N.M., Spuhler P.S., Fachin F., Lim E.J., Pai V., Ozkumur E., Martel J.M., Kojic N., Smith K., Chen P.I. (2014). Microfluidic, marker-free isolation of circulating tumor cells from blood samples. Nat. Protoc..

[bib45] Khoo B.L., Warkiani M.E., Tan D.S.W., Bhagat A.A.S., Irwin D., Lau D.P., Lim A.S.T., Lim K.H., Krisna S.S., Lim W.T. (2014). Clinical validation of an ultra high-throughput spiral microfluidics for the detection and enrichment of viable circulating tumor cells. PLoS One.

[bib46] Kim T.H., Wang Y., Oliver C.R., Thamm D.H., Cooling L., Paoletti C., Smith K.J., Nagrath S., Hayes D.F. (2019). A temporary indwelling intravascular aphaeretic system for in vivo enrichment of circulating tumor cells. Nat. Commun..

[bib47] Klein E.A., Richards D., Cohn A., Tummala M., Lapham R., Cosgrove D., Chung G., Clement J., Gao J., Hunkapiller N. (2021). Clinical validation of a targeted methylation-based multi-cancer early detection test using an independent validation set. Ann. Oncol..

[bib48] Ko J., Bhagwat N., Yee S.S., Black T., Redlinger C., Romeo J., O’Hara M., Raj A., Carpenter E.L., Stanger B.Z. (2017). A magnetic micropore chip for rapid (< 1 hour) unbiased circulating tumor cell isolation and in-situ RNA analysis. Lab Chip.

[bib49] Kuper S.W., Bignall J.R., Luckcock E.D. (1961). A quantitative method for studying tumour cells in blood. Lancet.

[bib50] Kwan T.T., Bardia A., Spring L.M., Giobbie-Hurder A., Kalinich M., Dubash T., Sundaresan T., Hong X., LiCausi J.A., Ho U. (2018). A digital RNA signature of circulating tumor cells predicting early therapeutic response in localized and metastatic breast cancer. Cancer Discov..

[bib51] Lambros M.B., Seed G., Sumanasuriya S., Gil V., Crespo M., Fontes M., Chandler R., Mehra N., Fowler G., Ebbs B. (2018). Single-cell analyses of prostate cancer liquid biopsies acquired by apheresis. Clin. Cancer Res..

[bib52] Lara O., Tong X., Zborowski M., Chalmers J.J. (2004). Enrichment of rare cancer cells through depletion of normal cells using density and flow-through, immunomagnetic cell separation. Exp. Hematol..

[bib53] Li P., Mao Z., Peng Z., Zhou L., Chen Y., Huang P.-H., Truica C.I., Drabick J.J., El-Deiry W.S., Dao M. (2015). Acoustic separation of circulating tumor cells. Proc. Natl. Acad. Sci. USA.

[bib54] Loutherback K., D’Silva J., Liu L., Wu A., Austin R.H., Sturm J.C. (2012). Deterministic separation of cancer cells from blood at 10 mL/min. AIP Adv..

[bib55] Martel J.M., Smith K.C., Dlamini M., Pletcher K., Yang J., Karabacak M., Haber D.A., Kapur R., Toner M. (2015). Continuous flow microfluidic bioparticle concentrator. Sci. Rep..

[bib56] Martel J.M., Toner M. (2014). Inertial focusing in microfluidics. Annu. Rev. Biomed. Eng..

[bib57] Micalizzi D.S., Maheswaran S., Haber D.A. (2017). A conduit to metastasis: circulating tumor cell biology. Genes Dev..

[bib58] Miller M.C., Robinson P.S., Wagner C., O’Shannessy D.J. (2018). The Parsortix^TM^ cell separation system—a versatile liquid biopsy platform. Cytometry.

[bib59] Mishra A., Dubash T.D., Edd J.F., Jewett M.K., Garre S.G., Karabacak N.M., Rabe D.C., Mutlu B.R., Walsh J.R., Kapur R. (2020). Ultrahigh-throughput magnetic sorting of large blood volumes for epitope-agnostic isolation of circulating tumor cells. Proc. Natl. Acad. Sci. USA.

[bib60] Miyamoto D.T., Lee R.J., Kalinich M., LiCausi J.A., Zheng Y., Chen T., Milner J.D., Emmons E., Ho U., Broderick K. (2018). An RNA-based digital circulating tumor cell signature is predictive of drug response and early dissemination in prostate cancer. Cancer Discov..

[bib61] Mutlu B.R., Dubash T., Dietsche C., Mishra A., Ozbey A., Keim K., Edd J.F., Haber D.A., Maheswaran S., Toner M. (2020). In-flow measurement of cell-cell adhesion using oscillatory inertial microfluidics. Lab Chip.

[bib62] Mutlu B.R., Smith K.C., Edd J.F., Nadar P., Dlamini M., Kapur R., Toner M. (2017). Non-equilibrium inertial separation array for high-throughput, large-volume blood fractionation. Sci. Rep..

[bib63] Nagrath S., Sequist L.V., Maheswaran S., Bell D.W., Irimia D., Ulkus L., Smith M.R., Kwak E.L., Digumarthy S., Muzikansky A. (2007). Isolation of rare circulating tumour cells in cancer patients by microchip technology. Nature.

[bib64] Ozkumur E., Shah A.M., Ciciliano J.C., Emmink B.L., Miyamoto D.T., Brachtel E., Yu M., Chen P.I., Morgan B., Trautwein J. (2013). Inertial focusing for tumor antigen-dependent and -independent sorting of rare circulating tumor cells. Sci. Transl. Med..

[bib65] Parkinson D.R., Dracopoli N., Petty B.G., Compton C., Cristofanilli M., Deisseroth A., Hayes D.F., Kapke G., Kumar P., Lee J.S.H. (2012). Considerations in the development of circulating tumor cell technology for clinical use. J. Transl. Med..

[bib66] Petersson F., Åberg L., Swärd-Nilsson A.M., Laurell T. (2007). Free flow acoustophoresis: microfluidic-based mode of particle and cell separation. Anal. Chem..

[bib67] Powell A.A., Talasaz A.H., Zhang H., Coram M.A., Reddy A., Deng G., Telli M.L., Advani R.H., Carlson R.W., Mollick J.A. (2012). Single cell profiling of circulating tumor cells: transcriptional heterogeneity and diversity from breast cancer cell lines. PLoS One.

[bib68] Reátegui E., van der Vos K.E., Lai C.P., Zeinali M., Atai N.A., Aldikacti B., Floyd F.P., Khankhel H.A., Thapar V., Sequist L.V., Hochberg F.H. (2018). Engineered nanointerfaces for microfluidic isolation and molecular profiling of tumor-specific extracellular vesicles. Nat. Commun..

[bib69] Reinhardt F., Franken A., Meier-Stiegen F., Driemel C., Stoecklein N.H., Fischer J.C., Niederacher D., Ruckhaeberle E., Fehm T., Neubauer H. (2019). Diagnostic leukapheresis enables reliable transcriptomic profiling of single circulating tumor cells to characterize inter-cellular heterogeneity in terms of endocrine resistance. Cancers.

[bib70] Riggi N., Aguet M., Stamenkovic I. (2018). Cancer metastasis: a reappraisal of its underlying mechanisms and their relevance to treatment. Annu. Rev. Pathol. Mech. Dis..

[bib71] Rodak B.F., Carr J.H. (2013).

[bib72] Sarioglu A.F., Aceto N., Kojic N., Donaldson M.C., Zeinali M., Hamza B., Engstrom A., Zhu H., Sundaresan T.K., Miyamoto D.T. (2015). A microfluidic device for label-free, physical capture of circulating tumor cell clusters. Nat. Methods.

[bib73] Saucedo-Zeni N., Mewes S., Niestroj R., Gasiorowski L., Murawa D., Nowaczyk P., Tomasi T., Weber E., Dworacki G., Morgenthaler N.G. (2012). A novel method for the in vivo isolation of circulating tumor cells from peripheral blood of cancer patients using a functionalized and structured medical wire. Int. J. Oncol..

[bib74] Stott S.L., Hsu C.H., Tsukrov D.I., Yu M., Miyamoto D.T., Waltman B.A., Rothenberg S.M., Shah A.M., Smas M.E., Korir G.K. (2010). Isolation of circulating tumor cells using a microvortex-generating herringbone-chip. Proc. Natl. Acad. Sci. USA.

[bib75] Stott S.L., Lee R.J., Nagrath S., Yu M., Miyamoto D.T., Ulkus L., Inserra E.J., Ulman M., Springer S., Nakamura Z. (2010). Isolation and characterization of circulating tumor cells from patients with localized and metastatic prostate cancer. Sci. Transl. Med..

[bib76] Trapani M.D., Manaresi N., Medoro G. (2018). DEPArray^TM^ system: an automatic image-based sorter for isolation of pure circulating tumor cells. Cytometry.

[bib77] Vermesh O., Aalipour A., Ge T.J., Saenz Y., Guo Y., Alam I.S., Park S.m., Adelson C.N., Mitsutake Y., Vilches-Moure J. (2018). An intravascular magnetic wire for the high-throughput retrieval of circulating tumour cells in vivo. Nat. Biomed. Eng..

[bib78] Wendel M., Bazhenova L., Boshuizen R., Kolatkar A., Honnatti M., Cho E.H., Marrinucci D., Sandhu A., Perricone P., Thistlethwaite P. (2012). Fluid biopsy for circulating tumor cell identification in patients with early-and late-stage non-small cell lung cancer: a glimpse into lung cancer biology. Phys. Biol..

[bib79] Werner S.L., Graf R.P., Landers M., Valenta D.T., Schroeder M., Greene S.B., Bales N., Dittamore R., Marrinucci D. (2015). Analytical validation and capabilities of the Epic CTC platform: enrichment-free circulating tumour cell detection and characterization. J. Circ. Biomarkers.

[bib80] Wong K.H.K., Edd J.F., Tessier S.N., Moyo W.D., Mutlu B.R., Bookstaver L.D., Miller K.L., Herrara S., Stott S.L., Toner M. (2018). Anti-thrombotic strategies for microfluidic blood processing. Lab Chip..

[bib81] Xu H., Aguilar Z.P., Yang L., Kuang M., Duan H., Xiong Y., Wei H., Wang A. (2011). Antibody conjugated magnetic iron oxide nanoparticles for cancer cell separation in fresh whole blood. Biomaterials.

[bib82] Yu M., Bardia A., Aceto N., Bersani F., Madden M.W., Donaldson M.C., Desai R., Zhu H., Comaills V., Zheng Z. (2014). Ex vivo culture of circulating breast tumor cells for individualized testing of drug susceptibility. Science.

[bib83] Yu W., Hurley J., Roberts D., Chakrabortty S.K., Enderle D., Noerholm M., Breakefield X.O., Skog J.K. (2021). Exosome-based liquid biopsies in cancer: opportunities and challenges. Ann. Oncol..

[bib84] Zhao M., Nelson W.C., Wei B., Schiro P.G., Hakimi B.M., Johnson E.S., Anand R.K., Gyurkey G.S., White L.M., Whiting S.H. (2013). New generation of ensemble-decision aliquot ranking based on simplified microfluidic components for large-capacity trapping of circulating tumor cells. Anal. Chem..

[bib85] Zheng G.X.Y., Terry J.M., Belgrader P., Ryvkin P., Bent Z.W., Wilson R., Ziraldo S.B., Wheeler T.D., McDermott G.P., Zhu J. (2017). Massively parallel digital transcriptional profiling of single cells. Nat. Commun..

